# High-quality draft genome sequence of *Paenibacillus* sp. RC80, a candidate for biofuel production

**DOI:** 10.1128/mra.01067-23

**Published:** 2024-06-07

**Authors:** Gabriella Rizzo, Mallory Choudoir, Rachel Simoes, Nipuni Dayarathne, Kristen M. DeAngelis

**Affiliations:** 1 Department of Microbiology, University of Massachusetts, Amherst, Massachusetts, USA; 2 Department of Plant and Microbial Biology, North Carolina State University, Raleigh, North Carolina, USA; University of Southern California, Los Angeles, California, USA

**Keywords:** soil microbiology, environmental microbiology, bioinformatics

## Abstract

*Paenibacillus* sp. RC80 was isolated from temperate deciduous forest soil in New England. The assembled genome is a single contig with 5,977,337 bp and 97.15% estimated completion. RC80 contains features for 2,3-butanediol dehydrogenase production and pathways involved in ethanol production.

## ANNOUNCEMENT

The identification of RC80 as a potentially novel species within the *Paenibacillus* genus hints at the vast reservoir of untapped soil microbial diversity, offering tantalizing prospects for biotechnological applications, particularly in the realm of biofuel production (1). Therefore, we sequenced a bacterial genome isolated from temperate forest soil (2) to understand the genetic profile of this novel species and its potential for biofuel production.

RC80 was isolated in 2022 from mineral soils collected, air-dried, and archived in 1998 from the Harvard Forest (2), a temperate, deciduous forest in Petersham, MA (42.54°N, 272.18°W). RC80 was isolated on ISP2 medium (3) and later grown on 10% tryptic soy agar (TSA), both at 25°C. For gDNA extraction, a single colony was grown in 10% tryptic soy broth (TSB) liquid media at 30°C, shaking at 150 rpm until an optical density of 0.5 was reached. Cells were pelleted using centrifugation at 4,000 rpm for 15 minutes, and genomic DNA was extracted using the cetyltrimethylammonium bromide (CTAB) method (4). The genome was sequenced on MinION (Oxford Nanopore Technology) using Nanopore R9 flow cells (R9.4.1) with SQK-LSK109 sequencing kit by SeqCenter (Pittsburgh, PA). High-accuracy base calling with Guppy v.4.5.4 was used to achieve Q20 performance. The DNA was not sheared or size-selected. The 16S rRNA gene was sequenced (4), and BLASTn (default settings) search (5) against the National Center for Biotechnology Information nucleotide database determined the closest cultured representative of RC80 to be *Paenibacillus polymyxa SC2* (98.8% identity).

The genome was assembled, annotated, and analyzed as part of the Bioinformatics Lab (MICROBIO 590B) course at University of Massachusetts Amherst (6). All software was deployed using default parameters unless otherwise specified. The raw read N50 for the Nanopore sequencing was 8,054 bp as determined by SeqFu (7). This generated 1,463,430,378 bp in 288,192 reads. The reads were subsampled to 40× target coverage (240,000,000 bp) and quality controlled using Filtlong v.0.2.1 (8). A *de novo* assembly was generated using Flye v.2.9.1 (9) followed by a consensus sequence using Minimap2 v.2.24 (10) and Racon v.1.4.3 (11).The final polishing was performed using Medaka v.1.7.2 (12). The genome was not trimmed, rotated, or circularized. Quality assessment of the sequence was evaluated using both QUAST v.4.4 (13) and CheckM v.1.0.18 (14). The annotation of the genome was completed using RAST v.1.073 (15) and Prokka v.1.14.5 (16) on Kbase (17). The final draft assembly contained one contig with an N50 of 5,977,337 bp, was estimated 97.15% complete and 0.07% contaminated, and contained nine copies each of 23S, 16S, and 5S ribosomal RNA genes and 71 tRNA copies. Therefore, the genome assembly is considered high quality (18). The average nucleotide identity (ANI) estimate (19) of *Paenibacillus polymyxa* SC2 was 85.43%. Since the ANI number is less than 95%, it is likely that this genome is a novel cultured species of *Paenibacillus*.

The RAST annotated genome shows that RC80 has 2,3-butanediol dehydrogenase alcohol forming feature. Additionally, RC80 is likely capable of producing ethanol because it has enzymes such as alcohol dehydrogenase present in the glycolysis pathway , determined using the Build Metabolic Model app v.2.0.0 (20). When produced in combination, 2,3-butanediol and ethanol make RC80 a potential candidate for biotechnological purposes, such as the production of biofuels (21).

## Data Availability

The static narrative for RC80 is available on KBase. The RAST and Prokka annotations are available on FigShare. The 16S rRNA gene sequence accession number for RC80 is OR690258.1. The raw whole genome sequence reads are available in GenBank under the BioProject accession number PRJNA1029665. The BioSample accession number for RC80 is SAMN38017374. The Sequence Read Archive (SRA) accession number for RC80 is SRR26593010. The complete genome reference number for RC80 is CP137860.
